# Optimizing Motor Coordination in Children with Developmental Coordination Disorder: Mini-Handball vs. Motor Skills Training

**DOI:** 10.3390/sports14010001

**Published:** 2025-12-29

**Authors:** Hurshida Bekmanova, Orifjon Saidmamatov, Jasurbek Jammatov, Taxirbek Salayev, Raximov Quvondiq, Shikhov Gayrat, Olga Vasconcelos, Rita Barros, Claúdia Sousa, Paula Rodrigues

**Affiliations:** 1 Faculty of Social and Applied Sciences, Urgench State Pedagogical Institute, Urgench 220100, Uzbekistan; 2 Faculty of Psychology and Social Sciences, Mamun University, Khiva 220100, Uzbekistan; saidorifjon@gmail.com (O.S.);; 3 Faculty of Sports Activities, Pedagogy and Psychology, Urgench State University, Urgench 220100, Uzbekistan; 4 Department of Taekwondo and Sports Activities, Mirzo Ulugbek National University of Uzbekistan, Tashkent 100174, Uzbekistan; 5 Motor Control and Learning Laboratory, CIFI2D, Faculty of Sport, University of Porto, 4200-450 Porto, Portugalpaula.rodrigues@ipiaget.pt (P.R.); 6 Insight: Piaget Research Center for Ecological Human Development, Av. João Paulo II, 1950-157 Lisboa, Portugal; rita.barros@ipiaget.pt (R.B.); claudia.sousa@ulusofona.pt (C.S.); 7 Centre for Research and Intervention in Education (CIIE) of the Faculty of Psychology and Education Sciences, Rua Alfredo Allen, 4200-135 Porto, Portugal; 8 HEI-Lab: Digital Human-Environment Interaction Lab, Lusofona University, 4000-098 Porto, Portugal

**Keywords:** developmental coordination disorder, motor skills training, mini-handball, MABC-2, sport-based intervention

## Abstract

Children with Developmental Coordination Disorder (DCD) experience motor competence challenges that hinder their participation in physical activities and affect daily functioning. While traditional motor skills training is commonly used, sport-based interventions offer the potential for greater benefits by providing dynamic, contextually rich environments for learning. This study aimed to evaluate the effectiveness of mini-handball training versus conventional motor skills training in improving coordination in children with DCD. Methods: Forty-four children aged 9–10 years from Khorezm, Uzbekistan, with coordination difficulties (scores below the 16th percentile in the MABC-2) were randomly assigned to three groups: mini-handball training (*n* = 15), motor skills training (*n* = 15), and control (*n* = 14). Both intervention groups participated in three 90 min sessions per week for 12 weeks. The mini-handball group engaged in sport-specific drills including passing, dribbling, shooting, and small-sided games, while the motor skills group performed balance, locomotor, and fine motor exercises. Pre- and post-intervention assessments were conducted using the MABC-2. Data were analyzed using linear mixed models with time, group, and their interaction as fixed effects. Results: Both intervention groups demonstrated significant improvements in motor coordination compared to controls. However, in general, the mini-handball group outperformed the other groups, particularly in domains requiring anticipatory control and visuomotor integration, including aiming and catching, balance, and overall coordination scores. Conclusions: Mini-handball represents a promising, ecologically valid intervention for children with DCD. By integrating motor skills practice with cognitive challenge, social interaction, and intrinsic motivation within a meaningful sport context, mini-handball appears more effective than traditional training approaches. These findings suggest that sport-based, open-skill interventions should be considered in therapeutic protocols, school curricula, and community programs for children with DCD. Future research should examine long-term retention, transfer to daily activities, and implementation across diverse populations.

## 1. Introduction

The neurodevelopmental disorder known as Developmental Coordination Disorder (DCD) is typified by notable deficiencies in the learning and application of coordinated motor skills. These deficits substantially hinder academic performance, everyday living activities, and general involvement [1], which, in turn, impacts children’s general development and social integration [2]. Children with DCD typically present clumsiness, delayed acquisition of motor milestones, and poor fine and gross motor control, often manifesting as difficulties with handwriting, object manipulation, or balance during sports and play activities. These deficits frequently lead to reduced physical activity levels, lower self-perceived competence, and social withdrawal compared with their typically developing peers [3,4,5]. The identification and improvement of motor-based interventions to help children with DCD improve their motor competence and functional outcomes have received more attention recently. Numerous methods have been investigated. In children with DCD, gross motor-based interventions—such as organized physical activities, body function exercises, group games, and small-group formats—have continuously shown promise in boosting motor function, increasing physical fitness, and preventing obesity. Additional proof of these interventions’ efficacy in enhancing gross motor function comes from a recent systematic review of research conducted between 2010 and 2022 [6].

Motor planning and predictive control issues are common in children with DCD, especially when it comes to tasks requiring a lot of time and space. Because they focus on the cognitive processes that underlie movement planning, interventions that use motor imagery and action observation have demonstrated promise in addressing these deficits [7].

Additionally, there is empirical support for cognitive-based interventions, particularly the Cognitive Orientation to Daily Occupational Performance (CO-OP) approach. This approach uses cognitive strategies to help children accomplish personally meaningful goals in daily activities, which improves their motor proficiency and occupational performance [7,8].

By utilizing peer interaction and motivating goal setting, group-based and goal-directed interventions have further proven their efficacy. These interventions, which usually combine motor learning principles, cognitive strategies, and structured practice schedules, are linked to notable enhancements in motor outcomes and participation in everyday activities [9].

The results of perceptual–motor interventions, which aim to improve the integration of sensory and motor processes, have been inconsistent. Although some research shows improvements in manual dexterity, balance, and object-control abilities like aiming and catching [10], other studies have not shown any appreciable progress [11].

Lastly, real-life tasks and targeted exercises are applied in activity-oriented and body function-oriented interventions to address specific motor deficits. These approaches have been shown to positively influence both motor function and skill acquisition [12,13]. A recent systematic review and meta-analysis of randomized controlled trials by Gao et al. [14] reported significant improvements in overall motor skills, balance, cognitive function, and activity performance following task-oriented interventions. Moreover, combining task- and process-oriented approaches has proven effective in enhancing general motor abilities in children with DCD.

Despite these various intervention options, there is limited evidence to help physical trainers, educators, and health professionals choose the most effective training approaches to improve physical fitness in children with poor motor coordination [15].

Recent research [16] has begun to investigate alternative intervention strategies that may provide broader benefits. Sports game interventions have shown promise in promoting the development of fundamental motor skills in children, implying that structured physical activities may offer a more engaging and contextually relevant approach to motor skills acquisition.

An example of this is mini-basketball, a sport that has proven effective in enhancing overall motor coordination in children with DCD [17]. Evidence indicates a close association between the development of complex motor skills and executive functions during childhood [18], implying that interventions integrating cognitively demanding activities may generate broader cognitive benefits. Within this context, mini-handball may be seen as a particularly promising modality for children with motor coordination difficulties. Previous studies highlight that mini-handball effectively promotes motor skills development, balance, and coordination [19], while its reliance on natural skills renders it accessible and easy to learn [20]. Furthermore, mini-handball is an inclusive adaptation of a team sport that encourages fair play, enjoyment, and physical literacy [21]. This perspective aligns with evidence showing that cognitively enriched physical activity, sports-based intervention models, and martial arts–based approaches produce meaningful improvements in balance, sensory integration, lower-limb strength, executive functioning, motivation, and overall motor proficiency in children with DCD, surpassing typical outcomes of standard physical education or non-adaptive motor practice [16,22,23,24,25]. Supporting this notion, a 12-week intervention study comparing open-skill (handball) and closed-skill (athletics) sports to a control group reported significant cognitive benefits associated with handball participation [26]. The dynamic demands of handball, requiring rapid decision-making, strategic planning, and motor adaptability, may, therefore, simultaneously foster motor and cognitive development [27].

Collectively, these findings highlight the importance of context-rich, cognitively engaging, and task-oriented intervention frameworks, as conventional fitness-based or repetitive practice approaches may be insufficient to drive neurofunctional and psychosocial adaptation [28].

However, empirical evidence directly comparing the effectiveness of mini-handball with traditional motor skills training in children with DCD is currently limited. Therefore, the present study aims to address this gap by conducting a comparative analysis of mini-handball and motor skills intervention programs in children with DCD, with the expectation—based on prior findings [17,28]—that mini-handball will lead to superior improvements in motor proficiency.

## 2. Methods

### 2.1. Participants

This study was conducted in Khorezm, Uzbekistan, with children aged 9–10 years. A group of 216 children (117 boys and 99 girls) was initially assessed using the Movement Assessment Battery for Children-Second Edition (MABC-2) tool to identify DCD. Based on the MABC-2 results, 129 children demonstrated scores below the 16th percentile, indicating coordination difficulties. Following the initial assessment, 85 children were excluded for several reasons. Children were not eligible if they presented with a diagnosed emotional, neurological, or movement disorder; a congenital, musculoskeletal, or cardiopulmonary condition that could influence postural control; intellectual impairment; current participation in physical or occupational therapy; regular or intensive sports training; disruptive behavior during screening; or difficulty understanding and following task instructions. Therefore, 35 children lived too far from the training site, creating a high risk of irregular attendance as reported by their parents; 25 were excluded due to medical contraindications or a history of prior rehabilitation; 20 did not submit written informed consent because the parents declined long-term participation; and 5 were excluded to avoid gender imbalance, as an equal distribution between boys and girls was required across study groups. Accordingly, after applying all inclusion and exclusion criteria, a total of 44 children were deemed eligible for enrollment. Then, they were randomly assigned to three subgroups: a mini-handball group (15 children: 13 scored below 5th percentile and 2 scored between 5th and 16th percentile), a motor skills training program group (15 children: 12 scored below 5th percentile and 3 scored between 5th and 16th), and a control group (14 children: 13 scored below 5th percentile and 1 scored between 5th and 16th percentile).

### 2.2. Instruments and Measures

The primary assessment instrument employed in this study was the Movement Assessment Battery for Children–Second Edition (MABC-2), which was administered before and after the intervention to evaluate coordination performance. All assessments were conducted by an assessor blinded to participant group allocation. Participants were initially assessed using the MABC-2 prior to randomization and then reassessed by the same blinded assessor after twelve intervention sessions or the designated control period.

The MABC-2 is considered an appropriate tool for assessing the development of motor competencies in preschool children, with acceptable evidence supporting its validity and reliability for Age Band 1 [29]. Smits-Engelsman et al. [12] investigated the psychometric properties of the MABC-2 in a sample of 50 typically developing three-year-old children and concluded, based on test–retest analyses, that the instrument demonstrates a satisfactory level of reliability, even at this young age, and is sensitive to detecting individual changes. Similarly, Ellinoudis et al. [12], in a study involving 183 Greek children assessed with Age Band 1, reported that the MABC-2 is a valid and reliable measure for identifying motor difficulties in children aged 3 to 5 years. The test consists of eight tasks grouped into three subscales: Manual Dexterity (MD) evaluates fine motor control and hand–eye coordination through tasks such as placing pegs, threading beads, and drawing within boundaries; Aiming and Catching (A-C) assesses visual–motor integration and coordination of upper-limb movements using activities involving throwing toward and catching targets; Balance (BAL) measures static and dynamic balance through tasks such as standing on one leg, walking along a straight line, or balancing on a board. Raw scores are converted to standardized scores for each subscale and summed to yield a total standard score, which reflects overall motor proficiency. According to the test manual [30], the following cutoff points are recommended: scores below the 5th percentile indicate DCD; scores between the 6th and 15th percentile indicate risk of DCD (r-DCD); and scores above the 16th percentile are indicative of typical development (TD). To ensure consistency, all pre- and post-intervention assessments were administered by the same examiner and recorded on video for subsequent verification.

### 2.3. Procedures

#### Study Design

This study employed a pre-test–post-test experimental design, consisting of two experimental groups and one control group.

Each experimental group participated in structured training sessions designed to enhance coordination abilities and overall motor skills. The intervention lasted 12 weeks, during which participants attended three 90 min sessions per week. Sessions were tailored to each group, with the mini-handball and motor skills training groups engaging in targeted exercises, while the control group received conventional physical education lessons.

The handball activities integrated fundamental and specific skills, supporting exploratory behaviors, perceptual–motor interactions, and adaptive learning. The sport-specific drills—including passing, dribbling, shooting, and small-sided games—aimed at improving hand–eye coordination, agility, and reaction time. Its playful, social, and motivating nature can also boost children’s intrinsic motivation and encourage lifelong physical activity habits, aligning with physical literacy goals. The training protocol was designed by a handball coach and organized into progressive stages: ball familiarization and grip; throwing; throwing and catching; combining throwing, catching, and movement; catching on the move; and dribbling.

The motor skills program included activities to develop manual dexterity, aiming and catching, and balance. Children manipulated small objects, threaded laces, and fastened buttons; caught and threw different balls; played bowling and reaction games; and practiced balance by walking on a line, picking up objects, responding to cues, and performing book-balancing tasks. The training protocol was designed by a trainer with a Ph.D. degree in the field of motor behavior and familiar with motor skills programs.

The control group continued to participate in their regular physical education classes as prescribed by the school curriculum, whereas only the two experimental groups were withdrawn from routine lessons and engaged in structured motor skills training sessions. The standard physical education classes consisted of general physical activities and traditional games, delivered in a broad, non-specialized format, without a specific focus on developing fundamental motor skills or sport-specific competencies.

All sessions were conducted by trained instructors following standardized protocols. Data were collected at baseline and post-intervention to evaluate the effectiveness of each program in improving coordination abilities, thereby allowing for comparative analysis across groups.

According to the Declaration of Helsinki, the research was approved by the Ethics Committee of Urgench State University (Code 12356).

### 2.4. Data Analysis

Descriptive statistics were calculated to describe all variables in this study. Four linear mixed models fit by maximum likelihood (one for each dimension and one for the total score of the MABC-2) were considered. According to Muth et al. [31] and Wiley and Rapp [32], linear mixed models are an adequate procedure for small sample designs. Time (pre-test, post-test) and groups (motor skills training, mini-handball, control) as well as their interaction were considered fixed effects. Subject-specific random intercepts were also considered to account for the repeated measurements. Assumptions were validated by analyzing the residuals graph. Following the guidelines of Meteyard and Davies [33], to decide between a model with only main effects and a model with main effects and interactions, for each dependent variable, nested models were considered and compared using Aikake Information Criterion (AIC), Bayesian Information Criterion (BIC), and Likelihood Ratio (LTR) tests. The main effects were firstly added to the model, and afterwards, the 2-way interaction was also considered. The model with the lowest AIC, the lowest BIC, and with a significant LTR test was selected. For each fixed effect, ANOVAs (analyses of variance) were then performed using Satterthwaite’s method to detect significant effects. Whenever the interaction between main effects was statistically significant, multiple comparisons with Holm’s correction and visual representations were considered.

All statistical analyses were performed using R software (version 4.4.0), with packages lme4 (version 1.1-35.3), lmerTest (version 3.1-3), performance (version 0.15.0), multcomp (version 1.4-26), lsmeans (version 2.30-0), and ggplot2 (version 3.5.1). A significance level of 0.05 was considered.

## 3. Results

Table 1 presents the descriptive statistics for all dimensions and total score of the MABC-2 during both pre- and post-tests.

According to Table 1, the present sample presented higher scores in the post-test for all variables.

To analyze the significance of these differences and the influence of groups (motor skills training, mini-handball, control) in these results, linear mixed models were considered.

Table 2 presents the linear mixed model selection for each of the variables in Table 1. Main fixed effects were first added to the model. Afterwards, the two-way interaction was also added. At each step, the model was compared to the previous one. The model with the lowest AIC, the lowest BIC, and with a significant LTR test was selected.

As can be seen in Table 2, according to AIC, BIC, and the LTR test, the model with the main effects and the two-way interaction as fixed effects is the one with the best performance for all dependent variables and will be the one considered in the following analyses.

### 3.1. Manual Dexterity

Table 3 presents the results of the linear mixed model, namely, the effect of each independent variable, with the results from ANOVAs performed using Satterthwaite’s method.

**Figure 1:**
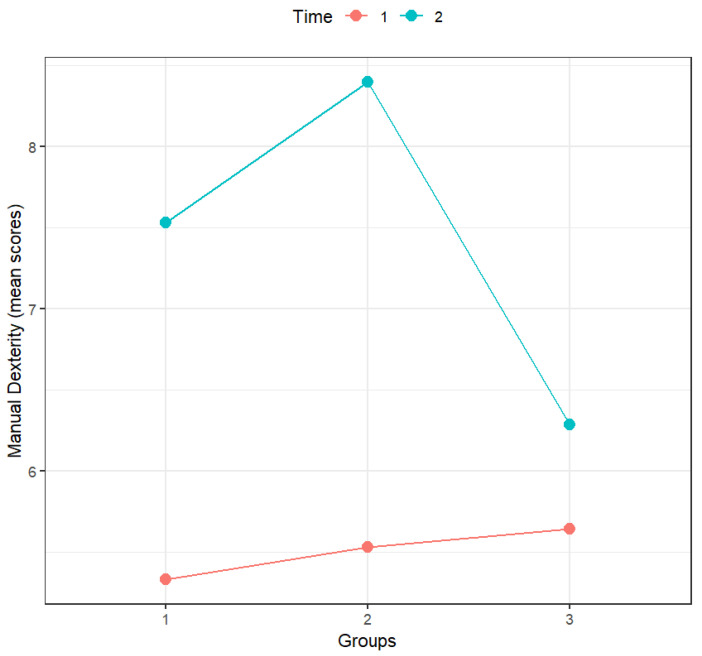
Effect of the interaction between groups and time on manual dexterity (*n* = 44). Notes: Time: 1—Pre-test, 2—Post-test; Groups: 1—Motor skills training (*n* = 15), 2—Mini-handball (*n* = 15), 3—Control group (*n* = 14).

According to Table 3, only time and the interaction between time and groups had a statistically significant effect on manual dexterity. As can be confirmed by analyzing Figure 1 and the significance of the multiple comparisons (with Holm’s correction; Table 4), in the pre-test, the scores of all groups were similar, but the scores in the post-test were always higher than those in the pre-test for all groups. However, in the control group, those differences between scores in the pre-test and post-test were not statistically significant.

When comparing the groups, the only statistically significant differences were the post-test difference between the mini-handball group and the control group, the change in scores from pre- to post-test in the motor skills training group, and the change in scores from pre- to post-test in the mini-handball group.

### 3.2. Aiming and Catching

Table 5 presents the results for the linear mixed model, namely, the effect of each independent variable, with the results from ANOVAs performed using Satterthwaite’s method.

**Figure 2:**
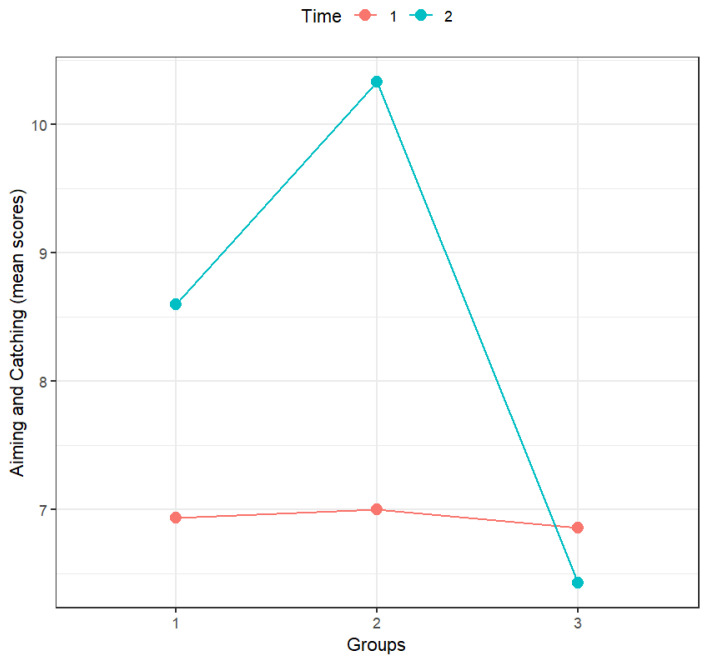
Effect of the interaction between groups and time on aiming and catching (*n* = 44). Notes: Time: 1—Pre-test, 2—Post-test; Groups: 1—Motor skills training (*n* = 15), 2—Mini-handball (*n* = 15), 3—Control group (*n* = 14).

According to Table 5, time, groups, and the interaction between time and groups had a statistically significant effect on aiming and catching. As can be confirmed by analyzing Figure 2 and the significance of the multiple comparisons (with Holm’s correction; Table 6), in the pre-test, the scores of all groups were similar, and the scores in the post-test were higher than those in the pre-test for the motor skills training group and mini-handball group but only significantly higher for the mini-handball group. The differences between pre- and post-tests for the control group were not statistically significant.

The interaction between time and groups was statistically significant. When comparing groups, the only statistically significant differences were the differences between the mini-handball group and the control group in the post-test as well as the difference in the mini-handball group scores between the pre- and post-test.

### 3.3. Balance

Table 7 presents the results for the linear mixed model, namely, the effect of each independent variable, with the results from ANOVAs performed using Satterthwaite’s method.

**Figure 3:**
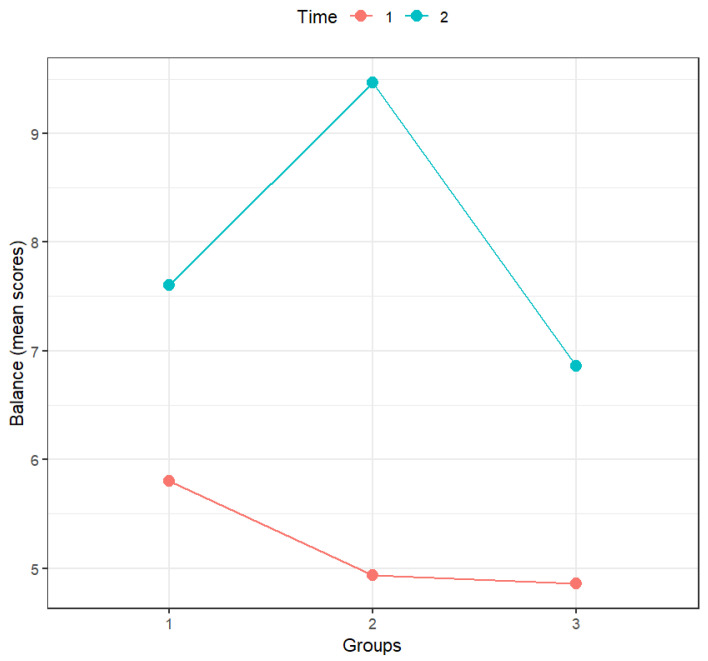
Effect of the interaction between groups and time on balance (*n* = 44). Notes: Time: 1—Pre-test, 2—Post-test; Groups: 1—Motor skills training (*n* = 15), 2—Mini-handball (*n* = 15), 3—Control group (*n* = 14).

According to Table 7, only time and the interaction between time and groups had a statistically significant effect on balance. As can be confirmed by analyzing Figure 3 and the significance of the multiple comparisons (with Holm’s correction; Table 8), in the pre-test, the scores of all groups were similar, and the scores in the post-test were always significantly higher than those in the pre-test for all groups. However, the difference between pre- and post-test scores was more accentuated for the mini-handball group.

The interaction between time and groups was statistically significant. When comparing groups, the only statistically significant differences were the differences between the mini-handball group and the control group in the post-test, the difference in the motor skills training group scores between the pre- and post-test, the difference in the mini-handball group scores between the pre- and post-test, and also the difference in the control group scores between the pre- and post-test.

### 3.4. Total Test Score

Table 9 presents the results for the linear mixed model, namely, the effect of each independent variable, with the results from ANOVAs performed using Satterthwaite’s method.

**Figure 4:**
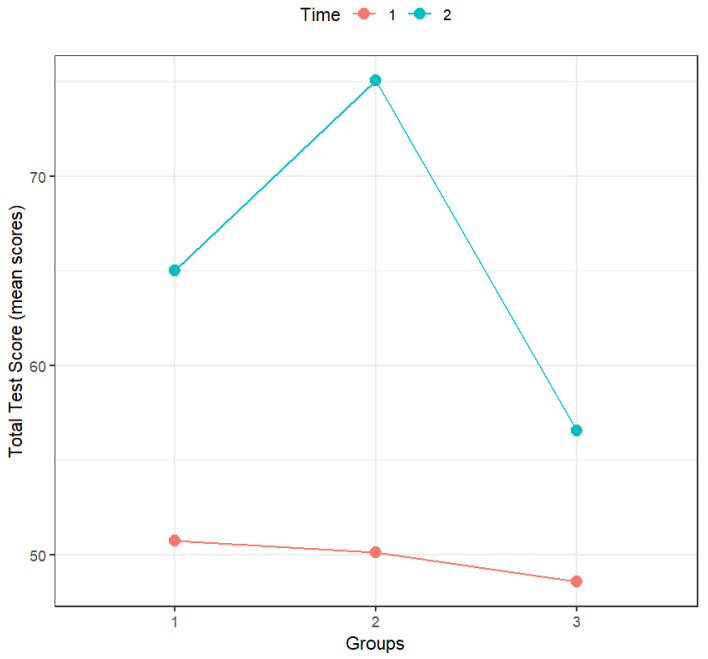
Effect of the interaction between groups and time on total test score (*n* = 44). Notes: Time: 1—Pre-test, 2—Post-test; Groups: 1—Motor skills training (*n* = 15), 2—Mini-handball (*n* = 15), 3—Control group (*n* = 14).

According to Table 9, time, groups, and the interaction between time and groups had a statistically significant effect on total test score. As can be confirmed by analyzing Figure 4 and the significance of the multiple comparisons (with Holm’s correction; Table 10), in the pre-test, the scores of all groups were similar, and the scores in the post-test were always significantly higher than those in the pre-test for all groups. However, the difference between pre- and post-test scores was more accentuated for the mini-handball group and less accentuated for the control group.

The interaction between time and groups was statistically significant. When comparing groups, there were statistically significant differences between all groups in the post-test and also when comparing each group’s scores between the pre- and post-test.

## 4. Discussion

The present study examined the comparative effects of mini-handball and traditional motor skills training on the coordination abilities of children with DCD. The findings suggest that mini-handball constitutes a highly effective approach for children with DCD, producing greater overall gains in the total test score than both conventional motor skills training and the control condition. These outcomes support the authors’ initial expectation that mini-handball would yield more substantial enhancements in motor competence. Concerning the three dimensions of the MABC-2 (manual dexterity, aiming and catching, and balance), both interventions (motor skills training and mini-handball) produced significant improvements in motor competence, while the control group showed minimal changes. Specifically, concerning manual dexterity, our results showed significant improvements for the motor skills training and mini-handball groups but not for the control group. Moreover, there were significant differences between the mini-handball and control groups, and the interaction between time and groups was significant. For aiming and catching, significant improvements were observed only in the mini-handball group, while no significant changes occurred in the motor skills training or control groups. Significant differences were also found between the mini-handball and control groups, and the interaction between time and groups was significant. Regarding balance, all three groups—motor skills training, mini-handball, and control—showed significant differences over time. There were also significant differences between the mini-handball and control groups, and the interaction between time and groups was significant. Finally, for the total score, significant differences were found across all groups. Significant differences were identified between every pair of groups (motor skills training vs. mini-handball, motor skills training vs. control, and mini-handball vs. control), and the interaction between time and groups was significant. For all the variables of the MABC-2 (manual dexterity, aiming and catching, balance, and total score), the interaction between time and group was significant. These findings highlight the potential of sport-based interventions, particularly mini-handball, as effective modalities for fostering motor development in children with DCD.

The present findings are consistent with prior research highlighting the efficacy of gross motor-based and structured activity interventions in improving motor proficiency and physical fitness among children with motor coordination difficulties [6,12]. However, the current study extends this body of literature by demonstrating that an open-skill, sport-based activity such as mini-handball may offer more pronounced benefits compared to traditional, closed-skill training approaches. These results corroborate those of Zolghadr et al. [17], who indicate that mini-basketball training has a greater positive impact on the overall motor coordination of children with DCD compared to general physical education programs. Identical results were found in a previous study from Tsai et al. [34], who examined the potential effects of a 10-week soccer training program on inhibitory control and neuroelectric functioning in children diagnosed with DCD and in typically developing children. Pre- and post-intervention assessments involved a visuospatial attention orienting task performed with the lower extremities, while concurrent recordings of event-related potentials were obtained. Following the intervention, the DCD-training group exhibited notable improvements in inhibitory control and a reduction in latency response. Overall, these findings suggest that participation in soccer training yields measurable benefits in both neuroelectric indices and behavioral performance for children with DCD. However, Hung and Pang [35], in a randomized controlled pilot intervention study, verified that group-based training produced similar gains in motor performance compared to individual-based training when studying 8 year-old children with DCD. In a very recent study, Valadi et al. [36] adopted an ecological approach, investigated the effectiveness of non-linear pedagogy interventions based on the constraint-led approach for both fundamental movement skills and selected handball sports skills, and evaluated their impact on typical children’s physical literacy and quality of life. The study demonstrated that both constraint-led approach protocols (non-linear fundamental movement skills and non-linear handball sports skills) effectively improved physical literacy and quality of life in children aged 8–10 years. The authors observed that enhancing physical literacy led to a better quality of life, with the non-linear fundamental movement skills protocol producing greater improvements. These findings highlight that applying ecological dynamics principles through non-linear pedagogy and affordance-rich environments can promote physical literacy and support long-term well-being in children. These findings reveal a trend opposite to that observed in our study, as the group that received the fundamental motor skills intervention demonstrated greater improvement in motor literacy compared to the group that participated in the handball-based intervention. However, it is important to note that the children in that study did not present DCD. A plausible explanation for this discrepancy may lie precisely in the profile of children with DCD. Their reduced participation in physical activity, diminished self-perceived competence, and increased social disengagement can be reversed in sport contexts, as sport activities tend to be more stimulating and can, therefore, enhance motivation and engagement—both of which are essential for acquiring specific and meaningful skills. The peer interaction fostered by sport activities, along with the enjoyment derived from game participation, appears to be particularly beneficial for children with DCD.

Regarding the absence of significant differences between the mini-handball group and motor skills training group in the dimensions of the MABC-2 (manual dexterity, aiming and catching, and balance), a plausible explanation is the possible overlap in the motor demands inherent to both interventions. Despite differing in format, the two programs may have targeted largely similar underlying abilities, including hand–eye coordination, balance, object manipulation, and postural control. Consequently, the sport-specific context of mini-handball may not have conferred an additional advantage of sufficient magnitude to be reflected in the MABC-2 subscale scores. Another reason may be contributed to the absence of differences between the two groups. Although the duration and intensity of the intervention may have been adequate to elicit within-group gains, they may have been insufficient to produce distinct differences between the groups. A 12-week program delivered three times per week typically supports measurable pre–post progress; however, such a timeframe may not allow one intervention modality to yield substantially greater improvements than the other. If both groups were subjected to a higher number of sessions, the accumulated volume of practice could generate statistically significant differences between the groups.

However, as mentioned above, in the total MABC-2 test score, the mini-handball group stood out significantly from the other two groups. This result is consistent with research emphasizing the role of open-skill sports in stimulating not only motor learning but also higher-order cognitive processes, such as executive function, decision-making, and attentional control [18,26]. The dynamic, unpredictable, and socially interactive nature of mini-handball appears to provide children with repeated opportunities to engage in rapid motor planning, spatial–temporal adjustments, and flexible coordination strategies—mechanisms that may explain its superior effects. Considering the results and arguments presented, our results confirm the initial hypothesis that a mini-handball-oriented intervention leads to better improvements in motor proficiency.

Another important contribution of this study concerns the motivational and ecological dimensions of mini-handball. Unlike conventional motor drills, which may be perceived as repetitive and less engaging, mini-handball integrates skill development within a meaningful, enjoyable, and socially interactive context. Enjoyment and peer interaction are key determinants of adherence, particularly in pediatric populations, and may partly explain the greater efficacy observed in this group. This finding resonates with the concept of physical literacy, which emphasizes lifelong engagement in physical activity through the development of competence, confidence, and motivation [21]. By fostering both motor competence and intrinsic motivation, mini-handball may promote sustainable engagement in physical activity beyond the intervention period, addressing one of the persistent challenges in DCD management. Mini-handball is a sport that really improves motor coordination, driving motor learning and motor transfer according to the ecological dynamics idea. Across the variations in the task and the environmental constraints (field size, rules, number of players), children explore solutions in context and interact with each other, challenging themselves. This supports perception–action coupling and better generalization and transfer to activities of daily living, play activities, physical education, and a variety of sports. General motor skills practice can still be important to surpass DCD [37,38], but collective sports give children with DCD the motivation, skill adaptability, and social participation opportunities that general skills alone cannot provide.

The findings also carry important implications for practice. For physical trainers, educators, and health professionals, the evidence suggests that integrating sport-based activities such as mini-handball into school curricula, community programs, or therapeutic protocols may provide an effective, inclusive, and engaging strategy to support children with DCD. Moreover, the comparative analysis highlights the need to move beyond traditional therapeutic models, considering ecologically valid interventions that align more closely with children’s real-world experiences and social environments.

Despite these promising results, several limitations should be acknowledged. The sample size, while adequate to perform the considered analyses, remains modest and limits generalizability. Although it was sufficient to detect several significant group differences, there is the potential for type II errors, and non-significant findings should be carefully interpreted. The intervention duration of 12 weeks, although sufficient to produce measurable changes, does not allow conclusions about long-term retention or transfer of skills to everyday contexts. Furthermore, reliance on the MABC-2 as the sole outcome measure restricts the scope of evaluation to motor coordination without capturing potential gains in executive function, psychosocial participation, or quality of life. Future research should, therefore, adopt a more multidimensional assessment framework, incorporating cognitive, emotional, and social outcomes as well as longitudinal follow-up designs to determine the sustainability of intervention effects. Additionally, comparative studies across different cultural, educational, and socioeconomic contexts are essential to evaluate the adaptability and scalability of mini-handball interventions.

## 5. Conclusions

The results obtained indicate that mini-handball is a particularly effective intervention for children with DCD, demonstrating superior effects in the total test score compared to traditional motor skills training and the control group. The authors confirmed the initial hypothesis that mini-handball will lead to superior improvements in motor proficiency. Although both intervention programs—mini-handball and motor skills training—fostered progress in motor competencies, mini-handball, in general, showed greater efficacy. The control group exhibited no relevant changes over the study period. Given the evidence presented, the authors suggest that interventions based on open-skill sports, such as mini-handball, should be incorporated into therapeutic protocols, community programs, and school curricula for children with DCD, as they represent more effective and meaningful alternatives to approaches centered solely on traditional motor exercises.

## Figures and Tables

**Table 1:** Descriptive analysis of the MABC-2 during pre- and post-tests (*n* = 44).

Subscales	Pre-Test		Post-Test	
	M (SD)	Range	M (SD)	Range
Manual Dexterity	5.50 (1.41)	2–8	7.43 (2.08)	4–13
Aiming and Catching	6.93 (2.13)	1–11	8.50 (2.55)	3–14
Balance	5.20 (2.38)	1–10	8.00 (1.90)	4–12
Total Test Score	49.84 (7.19)	32–62	65.75 (9.78)	40–89

Notes: MABC-2 = Movement Assessment Battery for Children-Second Edition.

**Table 2:** Model selection for each dependent variable (*n* = 44).

Dependent Variable	Model Name	Nested/Simpler Model	Fixed Effects Added	Random Effects	Model Fit		LRT Test Against Nested	
				Subjects	AIC	BIC	df	χ^2^
Manual Dexterity	Null	-	-	Intercepts	378.2	385.6		
	Main effects	Null	Time + Groups	Intercepts	353.9	368.8	3	30.26 ***
	2-way interaction	Main effects	Time × Groups	Intercepts	348.9	368.7	2	9.08 *
Aiming and Catching	Null	-	-	Intercepts	413.4	420.8		
	Main effects	Null	Time + Groups	Intercepts	398.1	412.9	3	21.31 ***
	2-way interaction	Main effects	Time × Groups	Intercepts	390.4	410.3	2	11.64 **
Balance	Null	-	-	Intercepts	420.3	427.7		
	Main effects	Null	Time + Groups	Intercepts	388.2	403.1	3	38.12 ***
	2-way interaction	Main effects	Time × Groups	Intercepts	381.9	401.7	2	10.29 **
Total Test Score	Null	-	-	Intercepts	687.6	695.0		
	Main effects	Null	Time + Groups	Intercepts	615.2	630.0	3	78.44 ***
	2-way interaction	Main effects	Time × Groups	Intercepts	596.5	616.3	2	22.71 ***

Notes: Time: 1—Pre-test, 2—Post-test; Groups: 1—Motor skills training (*n* = 15), 2—Mini-handball (*n* = 15), 3—Control group (*n* = 14); AIC = Aikake Information Criterion; BIC = Bayesian Information Criterion; LRT = Likelihood Ratio Test; * *p* < 0.05, ** *p* < 0.01, *** *p* < 0.001.

**Table 3:** Linear mixed model results for manual dexterity.

	Manual Dexterity		
Fixed Effects	F	df1; df2	*p*
Time	42.89	1; 44	<0.001
Groups	2.05	2; 44	0.141
Time × Groups	5.04	2; 44	0.011
Random Effects (subject—intercept)			
Variance	1.86		
SD	1.36		
N_subjects_	44		
Observations	88		
Marginal R^2^/Conditional R^2^	0.329/0.540		

Notes: Time: 1—Pre-test, 2—Post-test; Groups: 1—Motor skills training (*n* = 15), 2—Mini-handball (*n* = 15), 3—Control group (*n* = 14); *p*-values for fixed effects calculated using Satterthwaite’s approximations.

**Table 4:** Multiple comparisons for significant interactions when the dependent variable is manual dexterity (*n* = 44).

Difference	Estimate	SE	t	*p*
Time1 Groups1—Time2 Groups1	−2.20	0.50	−4.42	<0.001
Time1 Groups1—Time1 Groups2	−0.20	0.60	−0.33	1.00
Time1 Groups1—Time2 Groups2	−3.07	060	−5.10	<0.001
Time1 Groups1—Time1 Groups3	−0.31	0.61	−0.51	1.00
Time1 Groups1—Time2 Groups3	−0.95	0.61	−1.56	0.865
Time2 Groups1—Time1 Groups2	2.00	0.60	3.33	0.013
Time2 Groups1—Time2 Groups2	−0.87	0.60	−1.44	0.920
Time2 Groups1—Time1 Groups3	1.89	0.61	3.09	0.025
Time2 Groups1—Time2 Groups3	1.25	0.61	2.04	0.358
Time1 Groups2—Time2 Groups2	−2.87	0.50	−5.76	<0.001
Time1 Groups2—Time1 Groups3	−0.11	0.61	−0.18	1.000
Time1 Groups2—Time2 Groups3	−0.75	0.61	−1.23	1.000
Time2 Groups2—Time1 Groups3	2.76	0.61	4.51	<0.001
Time2 Groups2—Time2 Groups3	2.11	0.61	3.46	0.010
Time1 Groups3—Time2 Groups3	−0.64	0.52	−1.25	1.000

Notes: Time: 1—Pre-test, 2—Post-test; Groups: 1—Motor skills training (*n* = 15), 2—Mini-handball (*n* = 15), 3—Control group (*n* = 14); Holm’s correction for multiple comparisons was used.

**Table 5:** Linear mixed model results for aiming and catching.

	Aiming and Catching		
Fixed Effects	F	df1; df2	*p*
Time	12.37	1; 88	<0.001
Groups	7.20	2; 88	0.001
Time × Groups	6.22	2; 88	0.003
Random Effects (subject—intercept)			
Variance	4.13		
SD	2.03		
N_subjects_	44		
Observations	88		
Marginal R^2^/Conditional R^2^	0.315/0.315		

Notes: Time: 1—Pre-test, 2—Post-test; Groups: 1—Motor skills training (*n* = 15), 2—Mini-handball (*n* = 15), 3—Control group (*n* = 14); *p*-values for fixed effects calculated using Satterthwaite’s approximations.

**Table 6:** Multiple comparisons for significant interactions when the dependent variable is aiming and catching (*n* = 44).

Difference	Estimate	SE	t	*p*
Time1 Groups1—Time2 Groups1	−1.67	0.74	−2.25	0.218
Time1 Groups1—Time1 Groups2	−0.07	0.74	−0.09	1.000
Time1 Groups1—Time2 Groups2	−3.40	0.74	−4.58	<0.001
Time1 Groups1—Time1 Groups3	0.08	0.75	0.10	1.000
Time1 Groups1—Time2 Groups3	0.50	0.75	0.67	1.000
Time2 Groups1—Time1 Groups2	1.60	0.74	2.16	0.237
Time2 Groups1—Time2 Groups2	−1.73	0.74	−2.34	0.218
Time2 Groups1—Time1 Groups3	1.74	0.75	2.31	0.218
Time2 Groups1—Time2 Groups3	2.17	0.75	2.88	0.056
Time1 Groups2—Time2 Groups2	−3.33	0.74	−4.50	<0.001
Time1 Groups2—Time1 Groups3	0.14	0.75	0.19	1.000
Time1 Groups2—Time2 Groups3	0.57	0.75	0.76	1.000
Time2 Groups2—Time1 Groups3	3.48	0.75	4.61	<0.001
Time2 Groups2—Time2 Groups3	3.90	0.75	5.17	<0.001
Time1 Groups3—Time2 Groups3	0.43	0.77	0.56	1.000

Notes: Time: 1—Pre-test, 2—Post-test; Groups: 1—Motor skills training (*n* = 15), 2—Mini-handball (*n* = 15), 3—Control group (*n* = 14); Holm’s correction for multiple comparisons was used.

**Table 7:** Linear mixed model results for balance.

	Balance		
Fixed Effects	F	df1; df2	*p*
Time	56.93	1; 44	<0.001
Groups	2.82	2; 44	0.070
Time × Groups	5.79	2; 44	0.006
Random Effects (subject—intercept)			
Variance	2.98		
SD	1.73		
N_subjects_	44		
Observations	88		
Marginal R^2^/Conditional R^2^	0.411/0.543		

Notes: Time: 1—Pre-test, 2—Post-test; Groups: 1—Motor skills training (*n* = 15), 2—Mini-handball (*n* = 15), 3—Control group (*n* = 14); *p*-values for fixed effects calculated using Satterthwaite’s approximations.

**Table 8:** Multiple comparisons for significant interactions when the dependent variable is balance (*n* = 44).

Difference	Estimate	SE	t	*p*
Time1 Groups1—Time2 Groups1	−1.80	0.63	−2.86	0.044
Time1 Groups1—Time1 Groups2	0.87	0.72	1.21	0.797
Time1 Groups1—Time2 Groups2	−3.67	0.72	−5.12	<0.001
Time1 Groups1—Time1 Groups3	0.94	0.73	1.29	0.797
Time1 Groups1—Time2 Groups3	−1.06	0.73	−1.45	0.753
Time2 Groups1—Time1 Groups2	2.67	0.72	3.73	0.004
Time2 Groups1—Time2 Groups2	−1.87	0.72	−2.61	0.069
Time2 Groups1—Time1 Groups3	2.74	0.73	3.77	0.004
Time2 Groups1—Time2 Groups3	0.74	0.73	1.02	0.797
Time1 Groups2—Time2 Groups2	−4.53	0.63	−7.19	<0.001
Time1 Groups2—Time1 Groups3	−0.08	0.73	−0.11	0.917
Time1 Groups2—Time2 Groups3	−1.92	0.73	−2.64	0.069
Time2 Groups2—Time1 Groups3	4.61	0.73	6.33	<0.001
Time2 Groups2—Time2 Groups3	2.61	0.73	3.58	0.006
Time1 Groups3—Time2 Groups3	−2.00	0.65	−3.07	0.027

Notes: Time: 1—Pre-test, 2—Post-test; Groups: 1—Motor skills training (*n* = 15), 2—Mini-handball (*n* = 15), 3—Control group (*n* = 14); Holm’s correction for multiple comparisons was used.

**Table 9:** Linear mixed model results for total test score.

	Total Test Score		
Fixed Effects	F	df1; df2	*p*
Time	147.22	1; 44	<0.001
Groups	14.64	2; 44	<0.001
Time × Groups	14.46	2; 44	<0.001
Random Effects (subject—intercept)			
Variance	36.95		
SD	6.08		
N_subject_	44		
Observations	88		
Marginal R^2^/Conditional R^2^	0.682/0.729		

Notes: Time: 1—Pre-test, 2—Post-test; Groups: 1—Motor skills training (*n* = 15), 2—Mini-handball (*n* = 15), 3—Control group (*n* = 14); *p*-values for fixed effects calculated using Satterthwaite’s approximations.

**Table 10:** Multiple comparisons for significant interactions when the dependent variable is total test score (*n* = 44).

Difference	Estimate	SE	t	*p*
Time1 Groups1—Time2 Groups1	−14.27	2.22	−6.43	<0.001
Time1 Groups1—Time1 Groups2	0.60	2.40	0.25	1.000
Time1 Groups1—Time2 Groups2	−24.33	2.40	−10.12	<0.001
Time1 Groups1—Time1 Groups3	2.16	2.45	0.88	1.000
Time1 Groups1—Time2 Groups3	−5.84	2.45	−2.39	0.077
Time2 Groups1—Time1 Groups2	14.87	2.40	6.18	<0.001
Time2 Groups1—Time2 Groups2	−10.07	2.40	−4.19	<0.001
Time2 Groups1—Time1 Groups3	16.43	2.45	6.72	<0.001
Time2 Groups1—Time2 Groups3	8.43	2.45	3.45	0.006
Time1 Groups2—Time2 Groups2	−24.93	2.22	−11.23	<0.001
Time1 Groups2—Time1 Groups3	1.56	2.45	0.64	1.000
Time1 Groups2—Time2 Groups3	−6.44	2.45	−2.64	0.051
Time2 Groups2—Time1 Groups3	26.50	2.45	10.83	<0.001
Time2 Groups2—Time2 Groups3	18.50	2.45	7.56	<0.001
Time1 Groups3—Time2 Groups3	−8.00	2.30	−3.48	0.006

Notes: Time: 1—Pre-test, 2—Post-test; Groups: 1—Motor skills training (*n* = 15), 2—Mini-handball (*n* = 15), 3—Control group (*n* = 14); Holm’s correction for multiple comparisons was used.

## Data Availability

The raw data supporting the conclusions of this article will be made available by the authors on request.
